# Supplementary material for: Complexome profiling of the Chlamydomonas psb28 mutant reveals TEF5 as an early PSII assembly factor
Source: Plant Cell. 2025 Mar 17;37(6):koaf055. doi: 10.1093/plcell/koaf055 (PMC12164590; doi:10.1093/plcell/koaf055)
Supplement: koaf055_Supplementary_Data [file koaf055_supplementary_data.zip › psb28_interactive.zip!/plots/intensity/qualityPlots/peptides/LHCB7_Cre02.g110750.t1.2.html]

### Cre02.g110750.t1.2

Trivial name: LHCB7  
SeqCoverage [median]: 16.20  
Mass: 41.32 kDa  
  
 

| Sequence | MissedCleavage | Mass [Da] | wtIntensity | muIntensity | Score |
| --- | --- | --- | --- | --- | --- |
| ASQEPEGR | 0 | 872.40 | 0.00 | 785.18 | 93.37 |
| EQASFAK | 0 | 779.38 | 614.22 | 3555.25 | 90.71 |
| LPELQAEADKTR | 1 | 1369.72 | 910.64 | 11117.20 | 85.85 |
| QVQEQLEAK | 0 | 1071.56 | 754.17 | 2244.77 | 84.57 |
| QVQEQLEAKLPELQAEADKTR | 2 | 2423.27 | 0.00 | 1447.87 | 37.38 |
| SSEQPAPADAPETLTTR | 0 | 1769.84 | 4782.10 | 10504.11 | 149.30 |
| YQEAELLHAR | 0 | 1228.62 | 1917.58 | 16417.93 | 95.57 |
|  |  |  | 8978.7 | 46072.3 |  |
